# Prediction of vascular access stenosis: Blood temperature monitoring with the Twister versus static intra-access pressure ratio

**DOI:** 10.1371/journal.pone.0204630

**Published:** 2018-10-29

**Authors:** Yoo Jin Choi, Young-Ki Lee, Hayne Cho Park, Eun Yi Kim, Ajin Cho, Chaehoon Han, Sun Ryoung Choi, Hanmyun Kim, Eun-Jung Kim, Jong-Woo Yoon, Jung-Woo Noh

**Affiliations:** 1 Hemodialysis Center, Kangnam Sacred Heart Hospital, Seoul, Korea; 2 Department of Internal Medicine, Hallym University College of Medicine, Seoul, Korea; 3 Department of Radiology, Hallym University College of Medicine, Seoul, Korea; 4 Department of Internal Medicine, Sahmyook Medical Center, Internal Medicine, Seoul, Korea; University of Wisconsin, UNITED STATES

## Abstract

**Background:**

The Kidney Disease Outcomes Quality Initiative (KDOQI) guidelines recommend intra-access flow (Qa) measurement as the preferred vascular access surveillance method over static intra-access pressure ratio (SIAPR). Recently, it has become possible to perform Qa measurement during hemodialysis using thermodilution method called blood temperature monitoring (BTM) with the Twister device. The aim of this study was to investigate the correlation between Qa by BTM and SIAPR and to compare the performance of two tests in prediction of vascular access stenosis.

**Methods:**

The study was performed from January 2016 to November 2017 and included 97 patients with arteriovenous fistulas (AVF). Qa by BTM and SIAPR were simultaneously measured every 1~3 months with a total of 449 measurements during study period.

**Results:**

In our study population, mean age was 59.9±10.0 years and 61.9% were diabetes. The mean Qa obtained by BTM was 1186±588 mL/min. There was no correlation between Qa by BTM and venous SIAPR (r = 0.061, P = 0.196). Angiography identified 36 stenotic AVFs (37.1%) among the study subjects. They included 13 cases with only inflow stenosis, 6 with only outflow stenosis, and 17 with stenosis on both sides. Receiver-operating characteristic (ROC) curve analysis showed that Qa by BTM had higher discriminative ability to diagnose vascular access stenosis compared to SIAPR (P <0.001). The Qa less than 583 mL/min showed the highest diagnostic accuracy in vascular stenosis prediction.

**Conclusion:**

Intradialytic measurement of Qa by BTM showed better diagnostic power over venous SIAPR in prediction of vascular access stenosis.

## Introduction

The function of vascular access is very important for optimal management in hemodialysis (HD) patients. Insufficient flow by vascular access stenosis cause inadequate dialysis or access thrombosis if not identified and treated in a timely fashion. Angiography is the gold standard modality to identify and characterize stenotic vascular lesions, but it is expensive and invasive. Radiocontrast media may reduce residual renal function in HD patients. Therefore, several non-invasive assessment tools were developed to observe the flow, pressure or recirculation process in vascular access during HD.

The 2006 Kidney Disease Outcomes Quality Initiative (KDOQI) guidelines for vascular access recommended the intra-access flow (Qa) measurement as the preferred vascular access surveillance method over static dialysis venous pressure measurement [1]. Static intra-access pressure ratio (SIAPR) is the static intra-access pressure normalized to mean arterial pressure (MAP). The SIAPR method is based on the assumption that high SIAPR reflects low access blood flow associated with hemodynamically significant stenosis [2]. Several studies demonstrated that access blood flow measured by ultrasound dilution technique and venous pressure were not correlated [3, 4], and it is still controversial which method is better to assess venous stenosis [2, 5].

The temperature gradient method was described and validated as an access surveillance method in the previous studies [6–8]. It has become possible to perform access flow measurements during HD treatment using devices that can be integrated into the dialysis machine itself. Fresenius 5008 generators can use thermodilution method (blood temperature monitoring: BTM). This dilution method calculates blood flow from the recirculation values obtained from lines in normal and reverse positions. In addition, if we adopt a specialized device named Twister (Fresenius Medical Care) between the HD needle and the blood line, we can simplify the access blood flow determination by shortening the time needed to reverse the HD blood lines [9].

Despite the wide availability of SIAPR and Qa measurements during HD, there was no study directly comparing SIAPR and Qa by BTM. The aim of this study was to investigate the relationship between Qa by BTM and SIAPR, and to compare the performance of two tests in prediction of vascular access stenosis.

## Materials and methods

### Study design and population

From March 2016 to January 2018, a total of 97 HD patients with arteriovenous fistulas (AVF) were enrolled in the study from Hallym University Kangnam Sacred Heart Hospital (Seoul, Korea). All patients were 18 years old or older and had been receiving twice or thrice weekly HD through functioning vascular access. The demographic information and biochemical parameters were collected by study nurses. The Qa by BTM and SIAPR were simultaneously measured every 1~3 months for a total of 449 measurements during study period. This study complied with the Declaration of Helsinki and was approved by the institutional ethics committee of Kangnam Sacred Heart Hospital (IRB No. 2018-01-029).

### Access flow (Qa) measurements by BTM

BTM automatically adjusts the temperature of dialysate fluid according to the body temperature of each HD patient. By adjusting the temperature of the dialysis fluid artificially, the recirculation rate of blood can be measured. Using the BTM module of the 5008S machine manufactured by Fresenius Medical Care, the arterial and venous temperature values were measured as well as the recirculation value, and the vascular access flow was calculated by the temperature gradient method [6].

**Fig 1:**
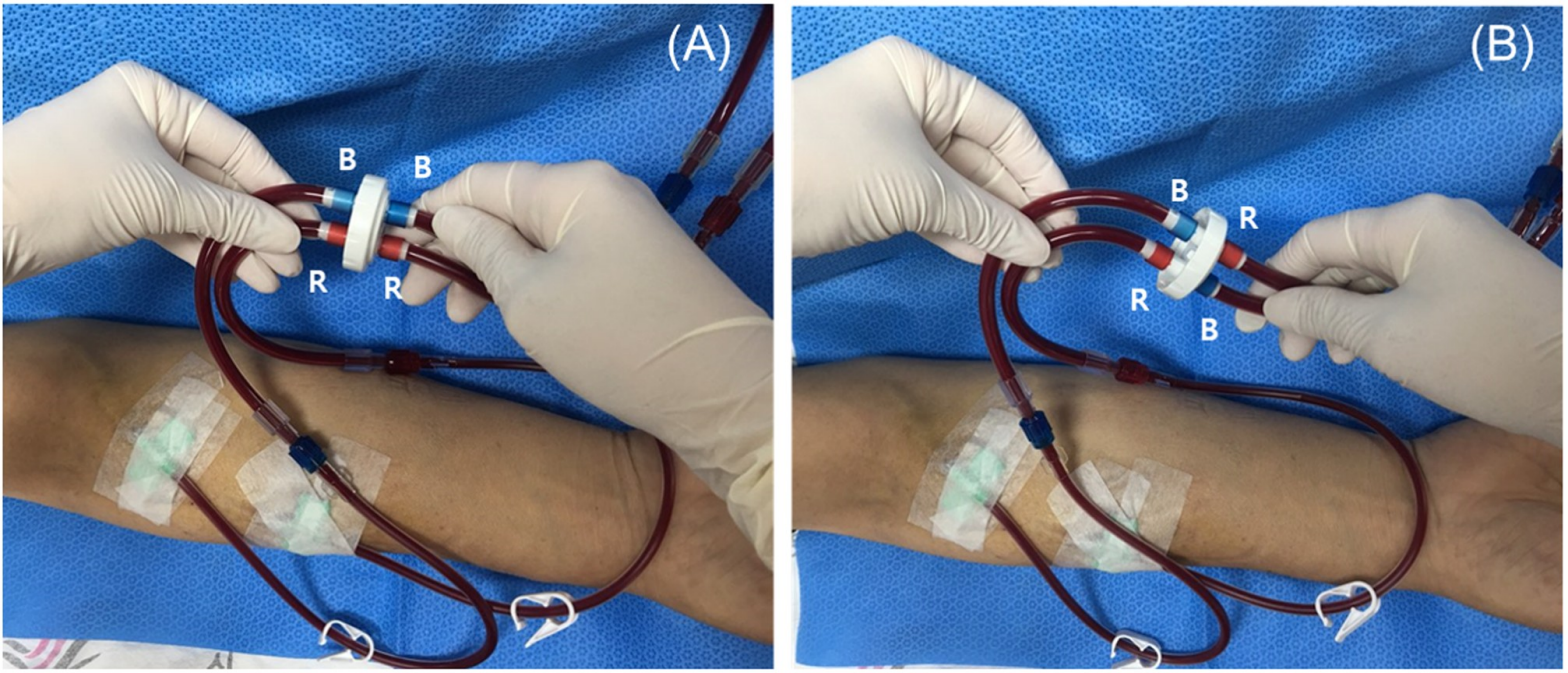
Twister device. (A) Twister device with the blood lines in the normal position. (B) Twister device with the blood lines in the reversed position after rotation.

The twister must be connected between the HD needles and the blood lines before HD begins (Fig 1). By simply rotating the dial halfway, the blood lines reverse flow direction automatically with no need for disconnecting them from the needles or stopping the HD pump [9]. There is no risk of blood exposure or infection as there is no need to separate the line from Twister while saving treatment time (Fig 1). BTM allows us to calculate the Qa from the temperature values obtained with the HD blood lines both in normal and inverted positions. The Qa was calculated using the following formula:

Qa=(QB.X-UFR)×Tart.x-Tven.xTart.n-Tart.x

$$Qa=\frac{\left(Q_{B.X}-UFR)\times [1-R_{X}-R_{N}+(R_{X}\times R_{N})\right]}{R_{X}-R_{X}\times R_{N}-\frac{\left(Q_{B.X}-UFR)\times (R_{N}-R_{X}\times R_{N}\right)}{Q_{B.N}}}$$

Q_B,N_: effective blood flow Q_Beff_ with regular tube connection (mL/min)

Q_B,X_: effective blood flow Q_Beff_ with reversed tube connection (mL/min)

UFR: ultrafiltration rate during recirculation measurement

R_N_: measured recirculation with regular tube connection (%)

R_X_: measured recirculation with reversed tube connection (%)

T_art,n_: temperature of the arterial line with blood lines in normal position

T_ven,x_: temperature of the venous line with blood lines in reverse position

### Static intra-access pressure ratio (SIAPR)

Venous SIAPR were measured in HD machines on which the pressure transducers were reset at atmospheric pressure prior to connection with the HD blood circuit [10]. Venous SIAPR measurements and calculations were made according to the KDOQI protocol [1] to eliminate the influence of the height between the needle and drip chamber, as well as mean arterial pressure (MAP).

SIAPR=(venouspressure+correctedvenousheight)/MAP

MAP=diastolicbloodpressure+〔(systolicbloodpressure−diastolicbloodpressure)/3〕

Correctedvenousheight=venousdripchamberupperpointofheight(cm)X0.76

### Prediction of venous access stenosis

All AVFs fulfilling the following inclusion criteria underwent angiography to evaluate the presence of a significant stenosis: (1) an access flow rate less than 400 mL/min in fistulae, (2) a decrease of 25% in access flow from a previous baseline value, (3) abnormal physical examination findings (abnormal thrill, bruit or pulse). Significant stenosis was defined as a decrease of greater than 50% of vessel diameter compared with the adjacent segment [11, 12] as ascertained by the radiologist who was unaware of the results of the other tests.

### Statistical analysis

Continuous variables were presented as the mean and standard deviations. Categorical variables were presented as numbers and percentages. Continuous variables were compared by independent t-test or Wilcoxon rank-sum test. Categorical variables were analyzed using the chi-square test. Pearson’s correlation analysis was performed to evaluate associations between Qa and SIAPR. Receiver-operating characteristic (ROC) curves were analyzed to determine overall screening accuracy of each test as measured by the area under the curve (AUC) and to identify optimal cut-offs for continuous variables. Differences between ROC curves were tested with the DeLong test. In addition, differences in diagnostic performance between tests and their optimal threshold(s) were assessed by comparing their sensitivity, specificity, positive predictive value (PPV), and negative predictive value (NPV) [13]. We used SPSS version 18.0 (IBM Corporation, Armonk, NY, USA). P values <0.05 was considered to be statistically significant.

## Results and discussion

Mean age of the patients was 59.9±10.0 years, and 50.5% were male. A history of diabetes mellitus was present in 61.9%. Table 1 summarized the most important clinical characteristics of the subjects. The mean Qa by BTM was 1186±588 mL/min (range: 166~3085 mL/min), and the mean values of SIAPR was 0.34±0.17. Patients with diabetes and forearm AVF showed significantly lower Qa (Table 2). However, other clinical variables did not significantly affect Qa.

**Table 1:** Characteristics of patients and AVF studied using BTM.

Gender (%)	men 49 / women 48
Age (years)	59.9±10.0 (range 22–83)
Diabetes mellitus	60 (61.9%)
HD duration (months)	71.8±66.9
Single-pool Kt/V	1.75±0.53
Site of AVF	forearm 84 / upper arm 13
Blood flow rate (mL/min)	264.8±14.6 (range 190–300)
Qa by BTM (mL/min)	1186±588 (range 166–3085)
SIAPR	0.34±0.17 (range 0.09–1.00)

AVF, arteriovenous fistulas; HD, hemodialysis; Qa, intra-access flow; SIAPR, static intra-access pressure ratio

**Table 2:** Relationship between the obtained intra-access flow and the variables.

Variable		No	Qa	P
Gender	Men	49	1184±688	0.166
	Women	48	1018±460	
Age	<60 years	48	1099±653	0.959
	≥60 years	49	1105±526	
Diabetes mellitus	(-)	37	1275±635	0.022
	(+)	60	996±537	
HD duration	<50 months	44	1077±633	0.698
	≥50 months	53	1124±555	
Single-pool Kt/V	<1.6	44	1120±667	0.805
	≥1.6	53	1090±526	
Site of AVF	Forearm	84	1025±503	0.031
	Upper arm	13	1602±844	
Blood flow rate	<270 mL/min	47	1110±526	0.893
	≥270 mL/min	50	1094±647	

AVF, arteriovenous fistulas; HD, hemodialysis

**Fig 2:**
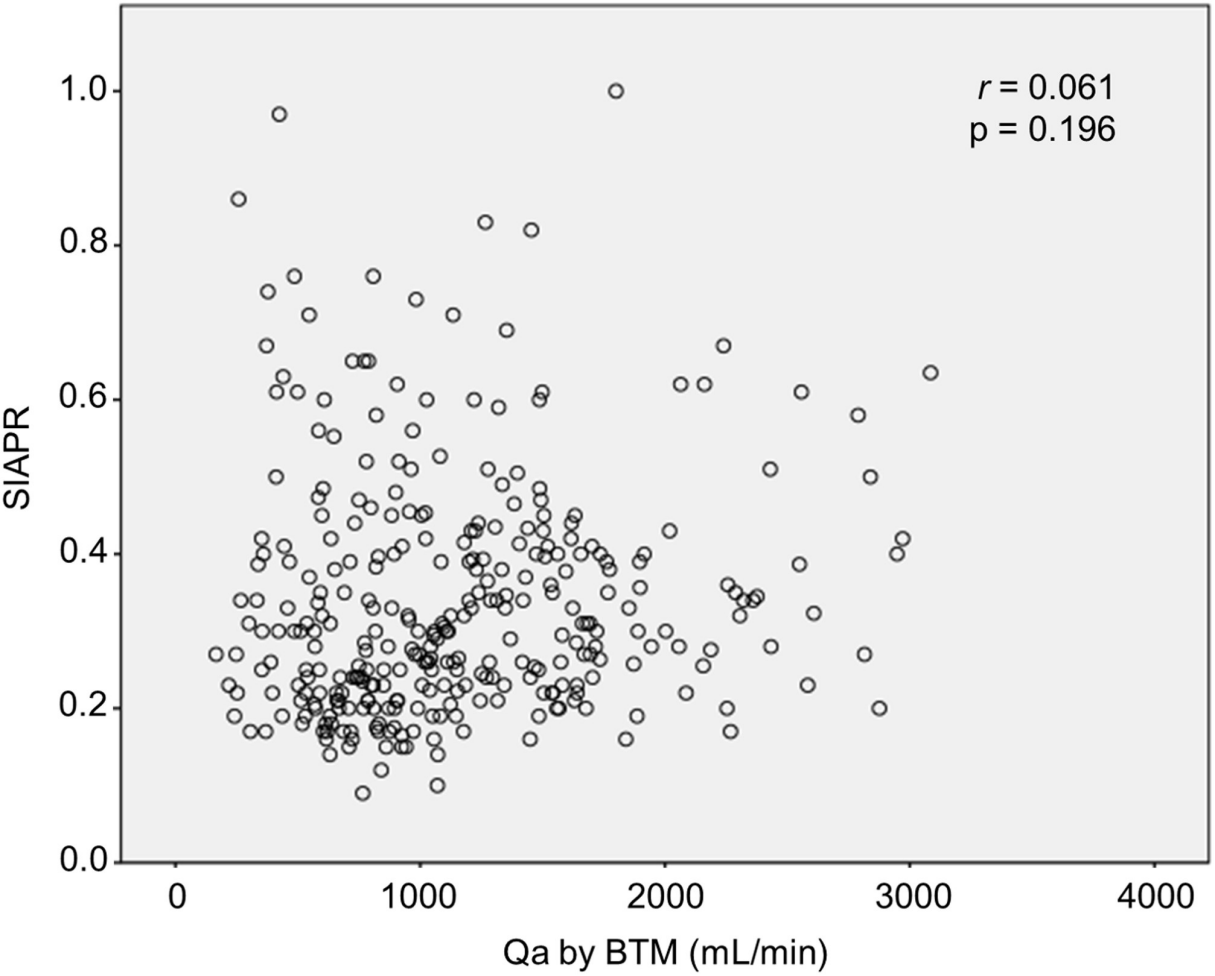
Access flow (Qa) measured by BTM and venous static intra-access pressure ratio (SIAPR).

There was no correlation between Qa by BTM and venous SIAPR (r = 0.061, P = 0.196, Fig 2). The Qa by BTM were similar between AVFs with abnormal SIAPR (according to KDOQI criteria: SIAPR >0.5) and normal SIAPR (1206±702 vs. 1182±562 mL/min, P = 0.776).

**Fig 3:**
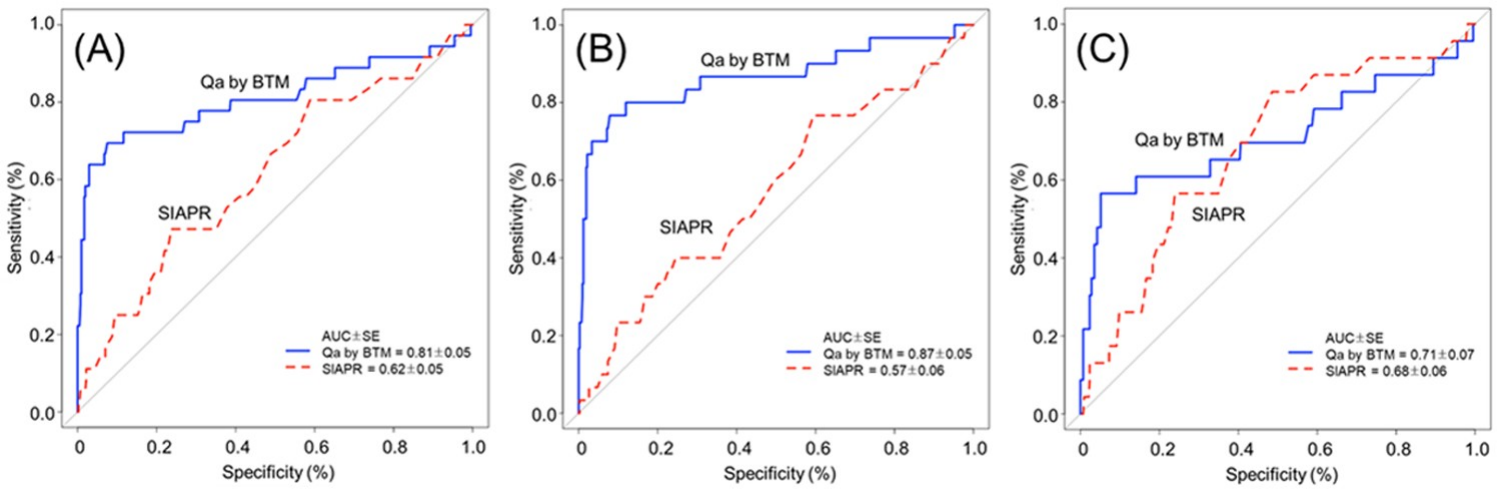
Diagnostic performance for stenosis at ROC curve analysis in arteriovenous fistulas. (A) Diagnostic performance of the tests for overall stenosis. (B) Diagnostic performance of the tests for inflow stenosis. (C) Diagnostic performance of the tests for outflow stenosis.

Angiography identified 36 stenotic AVFs (37.1%) in study subjects. There were 13 cases with only inflow stenosis, 6 with only outflow stenosis, and 17 with stenosis on both sides. For overall AVF stenosis, the diagnostic accuracy of Qa by BTM and SIAPR is shown in Fig 3. ROC curve analysis showed that Qa measurement by BTM had higher discriminative ability compared to SIAPR (AUC, 95% CI): 0.81 [0.71–0.91] vs. 0.62 [0.52–0.72], P <0.001, Fig 3A).

To compare diagnostic accuracy of each test by location of stenosis, we performed subgroup analysis in the case of inflow stenosis (30 in total, 13 only inflow stenosis and 17 both stenosis) and outflow stenosis (23 in total, 6 only outflow stenosis and 17 both stenosis). Especially in inflow stenosis, Qa measurement by BTM had higher discriminative power compared to SIAPR (P <0.001, Fig 3B). The AUC for Qa by BTM was 0.87 [0.78 to 0.96] (P <0.001), and for SIAPR it was 0.58 [0.46 to 0.69] (P = 0.175). But, Qa by BTM and SIAPR were equally moderate in their accuracy for outflow stenosis (Fig 3C). The AUC for Qa by BTM was 0.71 [0.57 to 0.86] (P = 0.001) whereas that for SIAPR was 0.68 [0.57 to 0.79].

Table 3 shows the diagnostic performance of each test for AVF stenosis. Qa thresholds of <500 and <600 mL/min seemed to have similar efficacy for detecting stenosis, but a threshold of <400 ml/min was associated with considerable loss of sensitivity without significant improvement in specificity. Qa <583 mL/min seems to be the most optimal threshold for predicting stenosis in patients with AVF. According to location of AVF, Qa <583 mL/min seems to be the optimal cut-off for predicting stenosis in the patients with forearm AVF whereas Qa <504 mL/min seems to be the most appropriate threshold in the patients with upper AVF.

**Table 3:** Diagnostic performance of the tests for AVF stenosis.

	Accuracy	Sensitivity	Specificity	PPV	NPV
Qa <300 mL/min	61.1% (50.1–72.1)	22.2% (11.1–36.8)	100%	100%	93.7% (92.8–94.8)
Qa <400 mL/min	71.4% (60.6–82.2)	44.4% (27.8–61.1)	98.3% (97.1–99.5)	70.0% (52.0–88.2)	95.3% (94.0–96.7)
Qa <500 mL/min	80.5% (70.8–90.2)	63.9% (50.0–80.6)	97.1% (95.4–98.6)	65.8% (52.5–81.3)	96.9% (95.6–98.3)
Qa <583 mL/min	81.0% (71.9–90.1)	69.4% (55.6–83.3)	92.5% (90.0–94.9)	44.6% (35.4–55.6)	97.2% (95.9–98.5)
Qa <600 mL/min	79.9% (70.8–89.0)	69.4% (55.6–83.3)	90.3% (87.4–93.0)	38.7% (30.3–48.2)	97.2% (95.8–98.4)
Qa <700 mL/min	78.2% (69.5–87.0)	72.2% (58.3–86.1)	84.3% (80.8–87.9)	28.7% (22.6–35.7)	97.3% (95.8–98.6)
SIAPR >0.5	55.9% (45.6–66.2)	30.6% (16.7–47.2)	83.5% (79.7–86.9)	13.9% (8.0–20.9)	93.2% (92.0–94.7)

PPV, positive predictive value; NPV, negative predictive value; Qa, intra-access flow; SIAPR, static intra-access pressure ratio

Our study confirms that SIAPR does not correlate with Qa by BTM and cannot discriminate between high and low Qa in AVFs. Our data also confirm that Qa by BTM is superior to SIAPR in prediction of AVF stenosis and is particularly useful for predicting inflow stenosis.

Access surveillance methods include monitoring intra-access blood flow, access recirculation and static dialysis venous pressure [14]. Each relies upon the observation that progressive stenosis increases SIAPR and decreases Qa. Unlike clinical monitoring, AVF surveillance program such as duplex ultrasound and ultrasonic dilution requires specialized equipment or specially trained staff and additional cost. Evidence suggests that Qa by BTM is fairly accurate in measuring access blood flow [8, 15, 16]. The advantage of BTM is that the need for a separate ultrasound dilution sensor and computer is no longer required. BTM is not operator-dependent and simultaneous measurements are possible during HD. In addition, the Twister device is useful to reduce the time for Qa by BTM [9].

Qa measurement is generally considered the most useful surveillance method. On the other hand, SIAPR is less expensive and easier surveillance method. Previous study by McCarley et al. compared Qa measurement by ultrasound dilution method and dynamic venous pressure monitoring and demonstrated that Qa method is superior than dynamic venous pressure monitoring [3]. Another study by Spergel et al. compared Qa measurement with SIAPR but demonstrated the lack of correlation between flow and pressure by the mathematical model [4]. To our knowledge, this is the first prospective study to demonstrate the superiority of Qa measurements by BTM over SIAPR.

The diagnostic performance of Qa by BTM was superior to that of venous SIAPR (P <0.001). In prediction of AVF stenosis, SIAPR had a lower sensitivity compared with Qa by BTM. Several studies have reported pressure surveillance to be ineffective and lacks predictive accuracy [4, 17].

In this study, inflow stenosis was detected with an excellent degree of accuracy by BTM (AUC 0.87 [0.78 to 0.96], P <0.001) and therefore Qa by BTM is suggested as the best initial screening procedure. However, Qa by BTM and venous SIAPR showed an equally diagnostic performance for outflow stenosis. Our results for inflow and outflow stenosis were similar to that from the study by Tessitore et al [18]. They demonstrated that optimal test for identifying an inflow stenosis was Qa <650 ml/min. Physical examination and both dynamic and derived static venous pressures >0.5 were equally highly diagnostic of outflow stenosis. Although the SIAPR method may detect outflow stenosis, it is likely to falsely target high-Qa or well-functioning accesses for referral, which may lead to unnecessary intervention for the best-functioning accesses [4]. SIAPR have a lower positive predictive value for stenosis in fistulas as compared with grafts [19]. Because SIAPR cannot predict vascular stenosis effectively, especially inflow stenosis, we consider SIAPR could not be routinely recommended in AVF.

Optimal thresholds of diagnostic tests were assessed by comparing their sensitivity and specificity. These data suggest that AVFs can be screened effectively for overall stenosis by measuring Qa alone considering a threshold of Qa <583 mL/min. The Qa threshold identified in our study is lower than that (Qa <750 mL/min) reported by Tessitore et al. [20] and higher than that (Qa <400 mL/min) reported by Lopot et al. [21]. According to location of AVF, Qa <583 mL/min seems to be the optimal cut-off value in forearm AVF while Qa <504 mL/min seems to be the most appropriate threshold in upper AVF. However, we cannot generalize our results and make a recommendation of Qa threshold for each anastomosis site because only 15 upper arm AVFs were included in our study. Moreover, we should not make clinical decision to undergo intervention only based on the Qa values since there may be some clinically insignificant stenoses. In the same vein, the KDOQI Guidelines recommend a vascular intervention when Qa of AVFs is <400–500 mL/min and such values are confirmed and correlated with clinical monitoring information [1].

We are aware that this study has some limitations. It is a single-center study on a small group of patients mainly with forearm AVFs and may be underpowered for the detection of some differences between the various tests. Our approach may also have limitations associated with subgroup analysis (forearm and upper arm AVF) since there were only 16 patients with upper arm AVFs in this study. In addition, our study did not apply other access surveillance methods such as ultrasound dilution techniques or Doppler ultrasound. Finally, we were not able to determine the optimal frequency of screening since patients were screened every 1 to 3 months depending on the vascular access status. Further prospective studies are warranted to demonstrate the clinical efficacy of routine vascular surveillance by BTM to improve thrombosis-related morbidity and associated costs. In addition, our findings should be validated in larger cohorts with not only AVFs but also AVGs.

## Conclusions

In conclusion, venous SIAPR neither correlated with Qa nor had a diagnostic power for stenotic AVFs. This study showed that AVF stenosis can be detected during HD with a moderate-to-excellent accuracy using Qa measurement by BTM as screening procedures.

## Supporting information

S1 File Raw data. (XLSX)
